# Effects of Cyclic Freeze–Thaw on the Steel Bar Reinforced New-To-Old Concrete Interface

**DOI:** 10.3390/molecules25051251

**Published:** 2020-03-10

**Authors:** Tao Luo, Chi Zhang, Xiangtian Xu, Yanjun Shen, Hailiang Jia, Chaowei Sun

**Affiliations:** 1 Shaanxi Key Laboratory of Safety and Durability of Concrete Structures, Xijing University, Xi’an 710123, China; luotao19870426@126.com (T.L.); zc595521884@163.com (C.Z.); chao_wei_106@126.com (C.S.); 2 Institute of Transportation, Inner Mongolia University, Hohhot 010070, China; 3 Geological Research Institute for Coal Green Mining, Xi’an University of Science and Technology, Xi’an 710054, China; shenyanjun993@126.com (Y.S.); hailiang.jia@xust.edu.cn (H.J.)

**Keywords:** new-to-old concrete, interface, shear strength, freeze–thaw cycles, reinforced

## Abstract

Frost damage of concrete has significant effects on the safety and durability of concrete structures in cold regions, and the concrete structures after repair and reinforcement are still threatened by cyclic freezing and thawing. In this study, the new-to-old concrete interface was reinforced by steel bar. The shear strength of the new-to-old concrete interface was tested after the new-to-old combination was subjected to cyclic freeze–thaw. The effects of the diameter of the steel bar, the compressive strength of new concrete, the number of freeze–thaw cycles and the freezing temperatures on the shear properties of new-to-old concrete interface were studied. The results showed that, in a certain range, the shear strength of the interface was proportional to the diameter of the steel bar and the strength of the new concrete. Meanwhile, the shear strength of the reinforced interface decreased with the decreasing of the freezing temperature and the increasing of the number of freeze–thaw cycles.

## 1. Introduction

The bond strength of the new-to-old concrete interface is an important index to evaluate the repair-and-strengthen effect for concrete structures [1,2]. Patnaik obtained a nonlinear expression of interfacial shear strength by interfacial shearing test of composite concrete beams [3]. Ray et al. made a new direct shear test apparatus to evaluate the bond capacity between high-performance concrete and normal concrete [4]. The bond strength can be affected by many factors, such as the roughness of the substrate concrete [5,6,7,8,9], the agent of the interface [10], the compressive strength of new concrete [11], the effect of the reinforcement [12], etc. The use of polypropylene fiber also effects the shear bond strength between self-compacting concrete and old concrete [13]. Different pretreatments on the surface of the old concrete can affect the bond strength significantly [14]; the effect of concrete substrate surface preparation can be characterized by roughness and micro-cracking in the near-to-surface layer [15].

The bearing performance of a concrete structure has a great relationship with the effect of environment [16,17,18]. In the northern part of China and the Qinghai–Tibet Plateau area, the temperature is less than 0 °C year-round, the concrete structure is vulnerable to freezing-and-thawing damage [19,20,21,22]. The mechanisms that govern frost behavior of concrete have been studied by many previous researches [23,24,25,26,27]. The harmful stresses caused by the damage of concrete can be concluded as: (a) hydraulic pressure caused by 9% of volume expansion during ice formation; (b) crystallization pressured induced by the growth of crystals in pores; and (c) the difference of thermal properties between the ice and cement matrix, etc.

After reinforcement and maintenance, the structure in the cold area still faces the threat of freezing-and-thawing damage, which requires the research on the antifreeze performance of the old and new concrete interface. Naderi pointed out that 300 freeze–thaw cycles could reduce the shear bond strength of a resin mortar by up to about 80%, and 200 cycles of temperature changes could reduce the original shear bond strength of a cementations mortar by up to about 90% [28]. The effect of cyclic freeze–thaw on the interface strength of new-to-old concrete was studied by Li et al. [29]. Their results showed that the interface shear strength of new-to-old concrete decreased sharply with the increasing of the number of freeze–thaw cycles. The degree of decline was influenced by the compressive strength of the new and old concrete and the characteristics of the interface, e.g., interface treatment method and property of interface agent.

The reinforcement of new-to-old concrete interface by anchorage is also used in the repair of concrete structures [12]. This study aimed at investigating the effect of cyclic freeze–thaw on the reinforced new-to-old concrete interface. The materials and experiments were introduced in Section 2. In Section 3, the test results were illustrated, and the effects of different reinforcement rates, different strengths of new concrete, the number of freeze–thaw cycles and different minimum freezing temperatures on the shear properties of new-to-old concrete interface were discussed and analyzed in detail. Some main conclusions were drawn in Section 4.

## 2. Results and Discussion

### 2.1. The Compressive Strength of Concrete Blocks

The cubic compressive test was carried out by using an MTS universal testing machine. The test procedure followed the Chinese Standard [30]. The compressive strength obtained for C30 and C35 concrete blocks are shown in Table 1. The average cubic compressive strengths for C30 and C35 were 32.3 and 36.4 MPa, respectively. Both the coefficients of variation for C30 and C35 were less than 15%; thus, these specimens were representative. 

### 2.2. The Shear Strength of New-To-Old Concrete Interface

**Figure 1:**
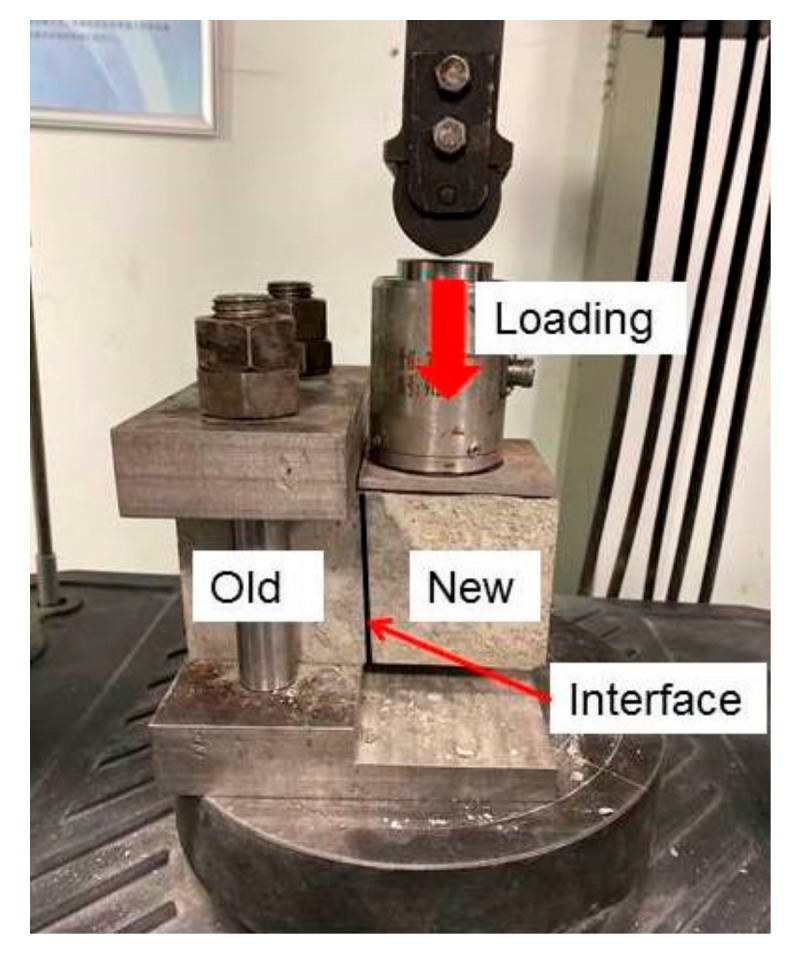
Shear fixture.

A shear fixture was developed for the interfacial shear test, which is shown in Figure 1. MTS universal testing machine was used for applying load. The interface shear strength could be calculated by the following equation:

(1) $$\tau =\frac{F}{A},$$

where τ is the interface shear strength, *F* is the largest shear force and *A* is the interface area.

The shear strengths of interfaces are shown in Table 2.

### 2.3. Failure Pattern of Reinforced New-To-Old Concrete Interface

**Figure 2:**
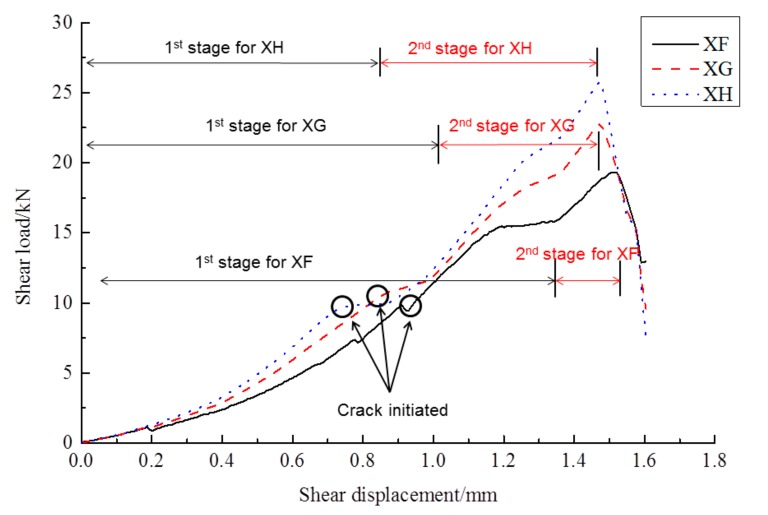
Shear load–displacement curves without frost damage.

**Figure 3:**
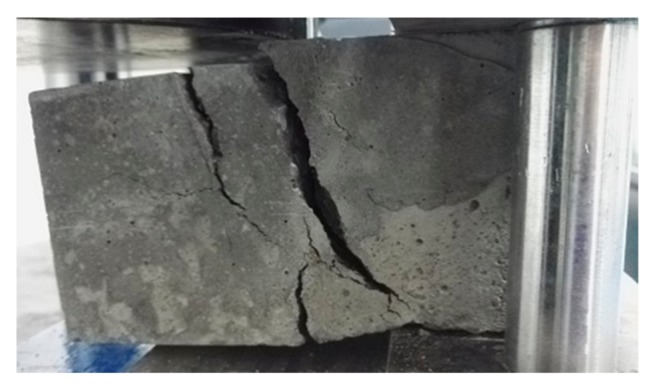
The failure mode of interface with the steel bar.

**Figure 4:**
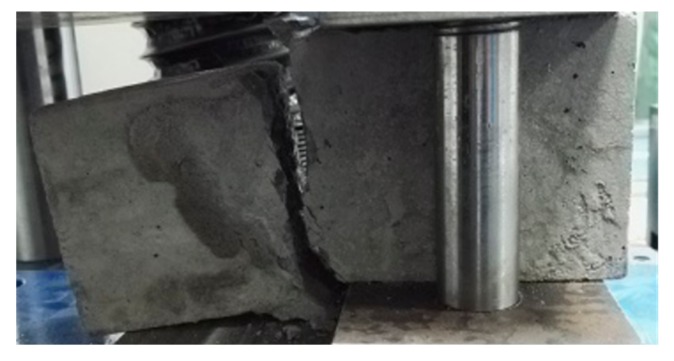
The failure mode of interface without the steel bar.

Figure 2 shows the shear load–displacement curves for interfaces reinforced by 8 mm (XF), 10 mm (XG) and 12 mm (XH), in the case of no frost damage. During the loading process, the reinforced new-to-old concrete interface experienced crack initiation, crack propagation and concrete-bond failure, which was defined as the first stage. At the end of the first failure stage, the interface staggered and opened up. As the loading continued, the shear force increased rapidly, and the steel bar undertook the main shear load, which was defined as the second stage. At the end of the second stage, a large number of cracks appeared in the concrete, and the peak state was achieved. Once the interface was totally broken, two sides of the upper part of the interface were pulled and separated, while the two sides of the lower part of the interface were squeezed together (Figure 3). The peak shear strength in the second stage was taken as the shear strength of the interface, but the phenomenon in the second stage might be partially caused by the bending stresses, which were neglected in this research. Figure 4 shows the failure mode of interface without the steel bar; the interface was separated, resulting in two concrete blocks without obvious cracks, which is quite different from Figure 3.

### 2.4. The Effect of Diameter of the Steel Bar on the Interface Shear Strength

A strength index, $\eta_{1}$, was used to compare the relative shear strength differences, which were defined as follows:

(2) $$\eta_{1}=\frac{\tau^{n}}{\tau^{nb}}\times 100,$$

where $\eta_{1}$ is the relative interface shear strength index, $\tau^{n}$ is the interface shear strength of new-to old concrete after n FTCs and $\tau^{nb}$ is the shear strength of concrete specimen without interface subjected to n FTCs.

**Figure 5:**
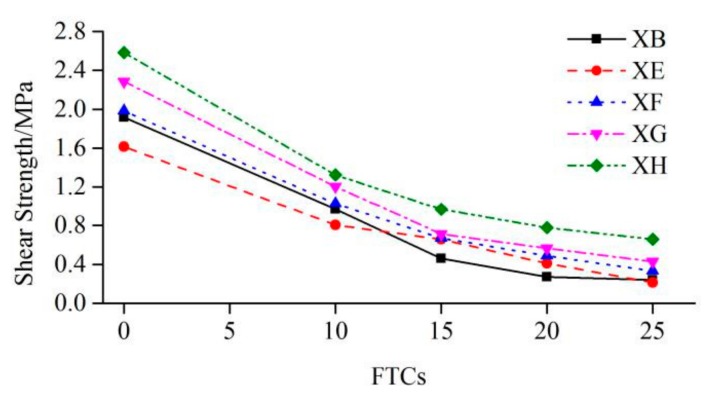
The shear strength versus FTCs.

Table 3 shows the values of $\eta_{1}$, and Figure 5 shows the shear strengths of interfaces under different number of FTCs. From Table 3 and Figure 5, under the same number of FTCs, the shear strengths of interfaces from small to large followed XE<XB<XF<XG<XH. With the increase of the diameters of steel bars, the shear strengths increased. With the increase of FTCs, the shear strengths of concrete specimens without interface decreased rapidly; the shear strengths of rough interfaces without the steel bar decreased rapidly in the early stage and then became smooth and rapid again at the end. While the shear strengths of interfaces with the steel bar decreased smoothly from 0 to 25 FTCs. The larger the steel bar was, the slower the shear strength decreased. With the increase of the number of FTCs, the improvement of the reinforcement was more effective. The larger steel bar had a better effect; for example, the shear strength of 12 mm steel-bar reinforced interface had been improved by 175%, with respect to the shear strength of concrete specimen without interface under 25 FTCs.

Another strength index, $\eta_{2}$, was used to consider the effect of compressive strength of concrete on the shear strength of interface, which was defined as follows:

(3) $$\eta_{2}=\frac{\tau^{n}}{\sigma^{nA}}\times 100,$$

where $\sigma^{nA}$ is the compressive strength of concrete cubic after n FTCs.

**Figure 6:**
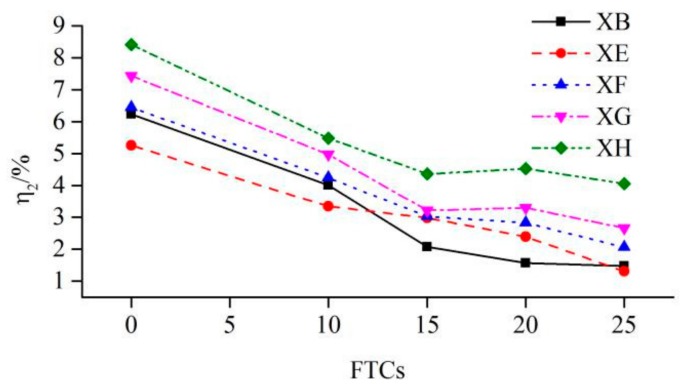
Curves of $\eta_{2}$ versus FTCs.

Table 4 shows the values of $\eta_{2}$. In Table 4, the values in the bracelets are compressive strengths of cubic concrete. With the increase of FTCs, the compressive strength of cubic concrete decreased monotonically. The values of $\eta_{2}$ in group XB were decreasing with the increasing of FTCs, meaning that the FTCs had a worse effect on the shear strength of concrete than the compressive strength of cubic concrete did. The relationship of $\eta_{2}$ and FTCs is shown in Figure 6. As shown in Table 4 and Figure 6, the values of $\eta_{2}$ in groups XE, XF, XG and XH were all decreasing with the increase of FTCs, meaning that the freeze–thaw resistance of the concrete body was better than the new-to-old concrete interface. The values of $\eta_{2}$ in all groups decreased rapidly before 15 FTCs, and then became relatively stable until 25 FTCs, indicating that the effect of FTCs on the compressive strength of concrete had a delay in respect to both the shear strength of the concrete body and the shear strength of the new-to-old concrete interface.

In order to study the effect of interface reinforcement by using the steel bar, strength index $\eta_{3}$ was introduced, which is defined as follows:

(4) $$\eta_{3}=\frac{\tau^{n}}{\tau^{nE}},$$

where $\tau^{nE}$ is the shear strength of coarse interface without steel bar reinforced after n FTCs.

**Figure 7:**
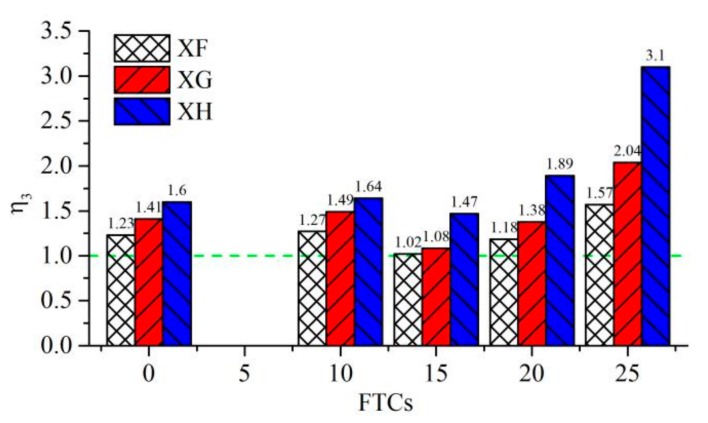
Values of $\eta_{3}$ under different FTCs.

The values of $\eta_{3}$ under different numbers of FTCs for groups XF, XG and XH are shown in Figure 7. All the values of $\eta_{3}$ were larger than 1, which means that the shear strength of interface was clearly strengthened by using the steel bar. After the new-to-old concrete interface was subjected to freeze–thaw cycles, the effect of reinforcement became more obvious. When FTCs reached 25, the shear strengths of interfaces reinforced by 8, 10 and 12 mm steel bars were 1.57, 2.04 and 3.1 times the shear strength of coarse interface without the steel bar, respectively.

### 2.5. The Effect of the Compressive Strength of New Concrete on the New-To-Old Concrete Interface

**Figure 8:**
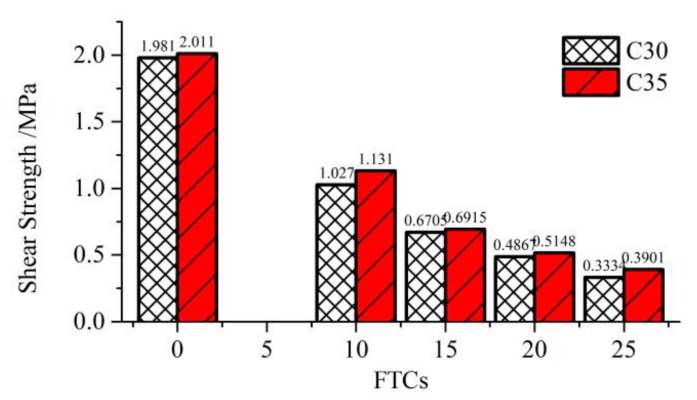
The shear strengths of interfaces by using C30 and C35 as the new concrete.

Two strength levels of C30 and C35 were used as new concrete to study the effect of the compressive strength of new concrete on the shear strength of the new-to-old concrete interface. The old concrete had the same compressive strength, and all the specimens were reinforced by an 8 mm steel bar. The shear strengths of interfaces for using C30 and C35 as new concrete are illustrated in Figure 8. The ratio of increased shear strength by using C35 as the new concrete is shown in Table 5.

From Figure 8 and Table 5, the shear strengths of interfaces by using C35 as new concrete were higher than using C30 as new concrete, which mean the shear strength of the interface could be increased by using new concrete with higher compressive strength. However, the effect was limited. The average increasing ratio of shear strength was 7.51%, which might be uneconomical in respect to the cost of using C35 rather than using C30.

### 2.6. The Effect of Freezing Temperature on the Shear Strength of New-To-Old Concrete Interface

Three different freezing temperatures, −6, −12 and −18 °C, were used to study the effect of minimum freezing temperatures on the shear strength of the new-to-old concrete interface. The thawing temperature was 5 °C, and the interface was reinforced by an 8 mm steel bar. These cases are grouped as XM, XK and XF in Table 4. All the new-to-old concrete combination specimens were subjected to 10, 15, 20 and 25 FTCs, respectively. An interfacial shear test was conducted once the FTCs reached the specific value. A strength index, $\eta_{4}$, was introduced to represent the residual shear strength rate of the interface after being subjected to different FTCs, and it is defined as follows:

(5) $$\eta_{4}=\frac{\tau^{n}}{\tau^{0}}\times 100,$$

where $\tau^{0}$ is the shear strength of new-to-old interface without cyclic freeze-thaw.

**Figure 9:**
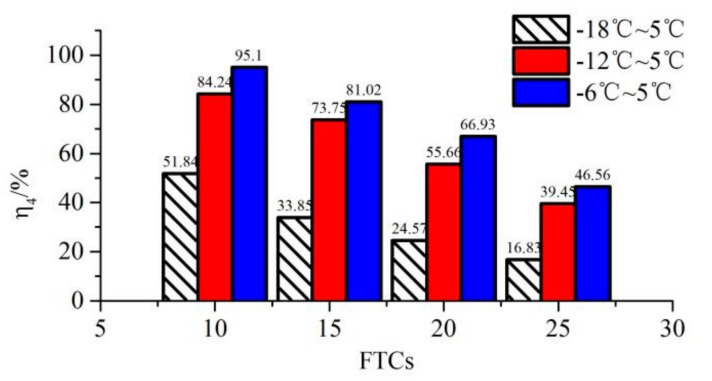
The residual shear strength rate of the interface after specific FTCs.

The residual shear strength rate of interface after specific FTCs is shown in Figure 9. For each specific FTC, the lowest freezing temperature had the smallest residual shear strength rate. For a freezing temperature of −18 °C, the residual shear strength rate was 51.84% after 10 FTCs, which was almost half the value of −6 °C after 10 FTCs. With the increase of FTCs, the residual shear strength rate was decreasing monotonically. For a freezing temperature of −18 °C, the residual shear strength rate decreased from 51.84% at 10 FTCs to 16.83% at 25 FTCs. Moreover, for a freezing temperature of −6 °C, the residual shear strength rate decreased from 95.1% at 10 FTCs to 46.56% at 25 FTCs. With the increase of FTCs, the lower freezing temperature had a more harmful effect on the shear strength of the new-to-old concrete interface.

## 3. Materials and Experiments

### 3.1. Materials

The Qinling P.O. 42.5 ordinary Portland cement produced by Shaanxi Yaoxian Cement Co., Ltd, Tongchuan, China. was used. The physical properties of Portland cement and its chemical compositions were detailed in Table 6 and Table 7, respectively.

Natural river sand with diameters between 0.5 and 1.17 mm was used as fine aggregate. Natural crushed stones with diameters between 5 and 20 mm were used as coarse aggregates. Sand and aggregates were cleaned, screened and dried before being used.

**Figure 10:**
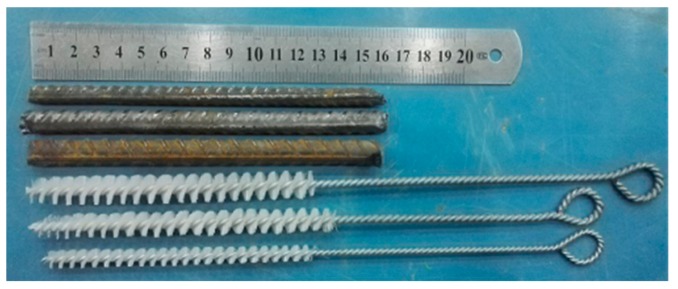
Steel bar for interface reinforcement.

Steel bars made of HRB400 (purchased from Shaanxi Steel Group Long Steel Company, Hancheng, China) were used for reinforcement, as shown in Figure 10. The diameters of the steel bars were 8, 10 and 12 mm, respectively, and the length of the steel bars was 160 mm. The reinforcement adhesive used in this study was produced by USA Aobang Group Hongkong New Type Building Materials Ltd., Hongkong, China.

### 3.2. Mix Composition

A strength level of C30 was used for old concrete, and two strength levels of C30 and C35 were used for new concrete. The dimensions of the cubic concrete block were 100 mm × 100 mm × 100 mm. The dimensions of the new-to-old concrete combination were 100 mm × 100 mm × 200 mm. Some concrete specimens with dimensions of 100 mm × 100 mm × 200 mm were used to compare with the new-to-old concrete combination. The mix composition of concrete is shown in Table 8.

### 3.3. Specimen Preparation and Experiments

Five different numbers of freeze–thaw cycles, 0, 10, 15, 20 and 25, were chosen for analyzing the effect of FTCs on the new-to-old concrete interface. Three specimens were prepared for each number of FTCs, and the results were taken from the average of these three specimens. Three different steel reinforcement diameters, 8, 10 and 12 mm, were used for studying the effect of reinforcement on the shear strength of new-to-old concrete interface. In order to study the effect of freeze temperature on the interface, −6, −12 and −18 °C were considered as three different minimum freeze temperatures. Based on the abovementioned, all the experimental cases were grouped into 8 groups. The details of the experimental scheme are shown in Table 9. The details of specimen preparation are described in the following.

**Figure 11:**
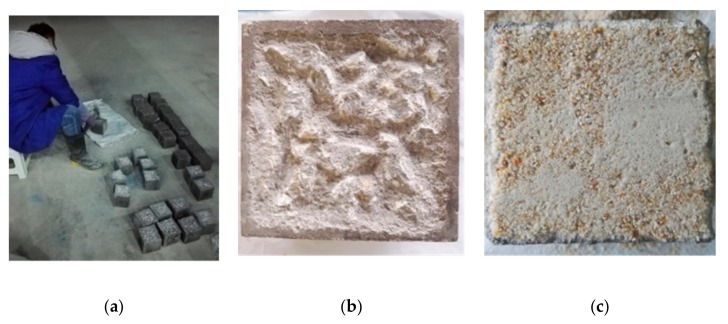
Rough-surface making and roughness measurement: (**a**) rough-surface making; (**b**) rough surface; and (**c**) sand filling.

(1) Mix composition of C30 was used for preparing 120 cubic concrete blocks. In total, 105 of them were prepared as old concrete for making new-to-old concrete combinations, and 15 of them were prepared for cyclic freeze-thaw tests.

(2) In total, 105 of the cubic concrete blocks were pretreated by making the surfaces rough, as shown in Figure 11a,b. The roughness was measured by a sand-filling method, as shown in Figure 11c. In the sand-filling method, the average sand-filling depth was adopted to represent the degree of roughness, which was defined as follows:

(6) $$\bar{h}=\frac{V_{sand}}{S_{oc}}$$

where *V*_sand_ is the volume of sand filled, and *S*_oc_ is the surface area of the old concrete.

The values of the roughness of the surfaces for old concrete are shown in Table 10. All the values are between 2 and 3 mm. The affection caused by the difference of roughness was neglected. 

**Figure 12:**
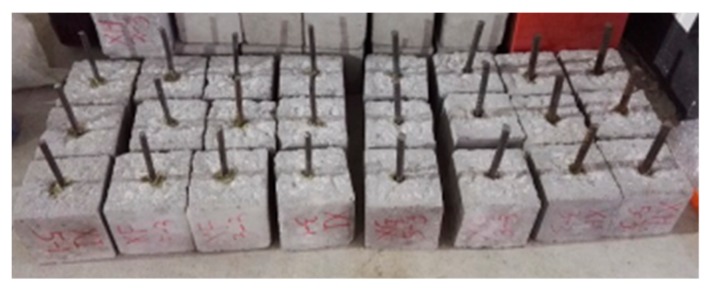
Reinforced old concrete blocks.

**Figure 13:**
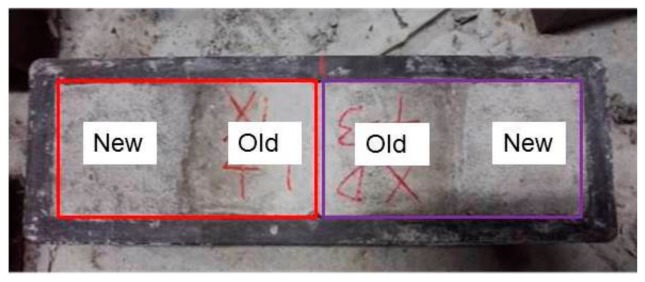
Two concrete combinations in one mold.

**Figure 14:**
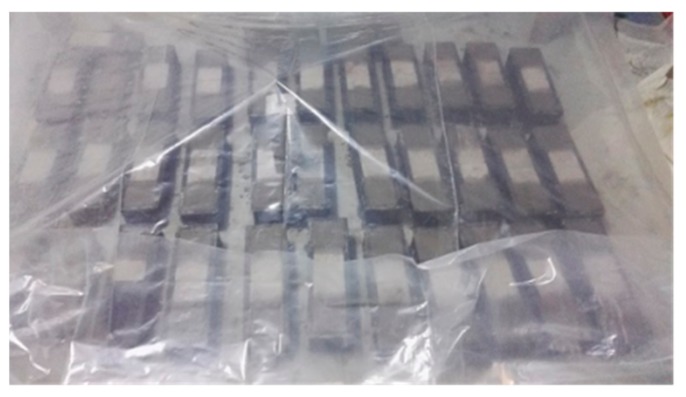
The specimens were cured for 28 days.

**Figure 15:**
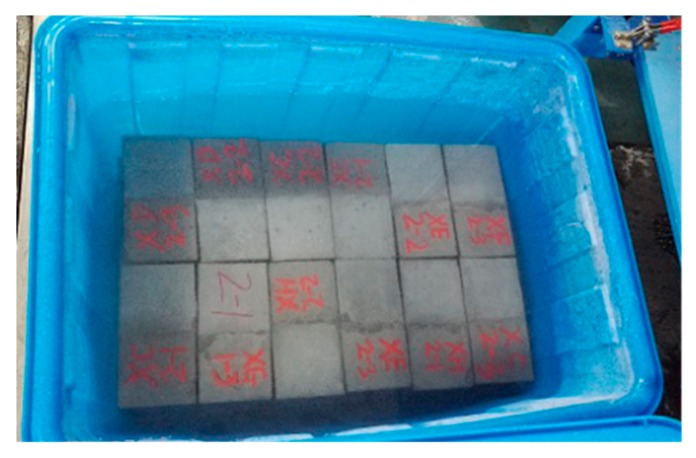
The concrete combinations were soaked for 4 days.

**Figure 16:**
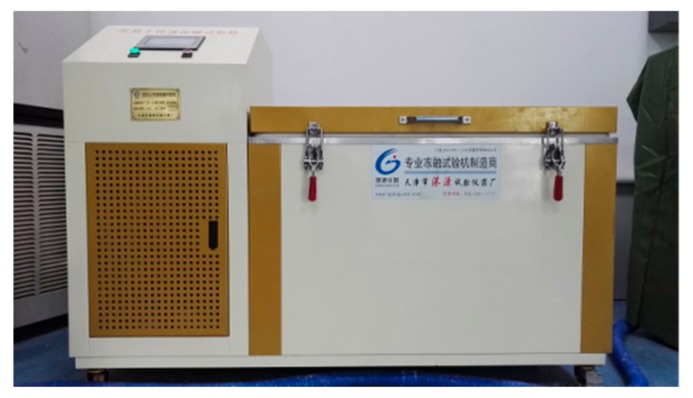
The rapid cyclic freeze-thaw test.

(3) The old concrete blocks were drilled after roughness was measured. Then the steel bar was put into the hole and glued with concrete, by reinforcement adhesive. The reinforced old concrete blocks are shown in Figure 12.

(4) The rough surfaces of the old concrete blocks were cleaned, presoaked and brushed with interface agent, for pouring new concrete. A mold with dimensions of 100 mm × 100 mm × 400 mm was used for making new-to-old concrete combinations. Each mold could fit two combinations. Two reinforced old concrete blocks were put into the mold first, and then new concrete was poured, as shown in Figure 13. Ninety C30–C30 new-to-old concrete combinations, 15 C30–C35 new-to-old concrete combinations and 15 C30 concrete cuboids with dimensions of 200 mm × 100 mm × 100 mm were prepared, and 3 C35 cubic concrete blocks were also made for cubic compression tests. All of these specimens were cured by covering a plastic membrane for 28 days, as shown in Figure 14. A cubic compressive test was conducted after the C30 and C35 concrete blocks were cured.

(5) The new-to-old concrete combinations were put into water and soaked for 4 days, when their ages reached 24 days. The water surface was kept 20 mm higher than the specimens, as shown in Figure 15. Four days later, all the specimens were taken out from the water and wiped with a wet rag. Then, we proceeded with the rapid cyclic freeze–thaw test (Figure 16). One cycle of freeze–thaw tests for the range of −18~5 °C, −12~5 °C and −18~5 °C took 8, 6 and 4 h, respectively. Once the number of FTCs reached 10, 15, 20 and 25, specimens were taken out for interface shear test.

## 4. Conclusions

Fifteen cubic concrete specimens, 15 concrete cuboids and 105 new-to-old concrete combinations were prepared for studying the effect of cyclic freeze–thaw on the shear strength of new-to-old concrete interfaces. All the specimens were subjected to 0, 10, 15, 20 and 25 FTCs, respectively. No interface, a rough interface without reinforcement and a rough interface reinforced by an 8, 10 and 12 mm steel bar were compared. Two different new concrete compressive strength levels, C30 and C35, were also considered. The influence of three different freezing temperatures, −6, −12 and −18 °C, on the shear strength of new-to-old concrete interfaces was studied. By the results of shear test of interface and analysis, the main conclusions could be drawn as follows:

(1) With the increase of FTCs, the shear strengths of both the concrete body and the new-to-old concrete interfaces were decreasing monotonically. The effect of FTCs on the compressive strength of concrete cubic had a delay in respect to both the shear strength of the concrete cuboid and the shear strength of new-to-old concrete interface.

(2) The shear strength of interface was clearly improved by using a steel bar. After the new-to-old concrete interface was subjected to freeze–thaw cycles, the reinforcement effect became more obvious. With the increase of the diameter of the steel bar, the shear strengths of interfaces were increasing. With the increase of FTCs, the improvement of the reinforcement was more effective, and the larger steel bar had a better effect.

(3) The shear strength of interface could be increased by using new concrete with higher compressive strength. However, the effect was limited. The economic cost should be considered in practical engineering repairing.

(4) Under the same FTCs, the lowest freezing temperature had the smallest residual shear strength rate. With the increase of FTCs, the lower freezing temperature had a more harmful effect on the shear strength of the new-to-old concrete interface.

Overall, the use of a steel bar as anchorage for the repair of concrete structures could improve the bearing capacity and frost resistance of a new-to-old concrete interface. Only one steel bar was used in this research; however, different numbers of steel bars could be studied in the future for guiding practical usage.

## Figures and Tables

**Table 1:** Cubic compressive strength for C30 and C35.

Group	No. of Specimen	Uniaxial Compressive Strength/MPa	Average Strength/MPa	Standard Deviation	Coefficient of Variation (%)
C30	1	32.2	32.3	0.07	0.22
	2	32.3			
	3	32.3			
C35	1	37.0	36.4	0.51	1.40
	2	36.3			
	3	36.0			

**Table 2:** The shear strengths of interfaces.

Group Name	Number of FTCs	Shear Strength/MPa	Coefficient of Variation (%)	Group Name	Number of FTCs	Shear Strength/MPa	Coefficient of Variation (%)
XB	0	1.92	0.98	XE	0	1.62	0.65
	10	0.97	1.34		10	0.81	0.87
	15	0.46	2.21		15	0.66	1.32
	20	0.27	1.98		20	0.41	2.36
	25	0.24	3.55		25	0.21	4.75
XF	0	1.98	2.48	XG	0	2.28	2.47
	10	1.03	3.22		10	1.20	3.26
	15	0.67	1.89		15	0.71	2.78
	20	0.49	4.32		20	0.57	1.58
	25	0.33	3.55		25	0.43	4.23
XH	0	2.58	2.35	XI	0	2.01	0.98
	10	1.32	3.21		10	1.13	2.31
	15	0.97	2.58		15	0.69	2.56
	20	0.78	4.23		20	0.51	1.97
	25	0.66	2.64		25	0.39	3.26
XK	0	1.80	3.31	XM	0	1.88	3.33
	10	1.52	2.14		10	1.78	2.69
	15	1.33	2.64		15	1.46	2.41
	20	1.00	2.58		20	1.21	4.12
	25	0.71	4.12		25	0.84	4.56

**Table 3:** The values of $\eta_{1}$.

Concrete Group	$\mathrm{Strength}\;\mathrm{Index}\;\eta_{1}$				
	0 FTCs	10 FTCs	15 FTCs	20 FTCs	25 FTCs
XB	100%	100%	100%	100%	100%
XE	84%	83%	144%	152%	89%
XF	103%	106%	146%	180%	139%
XG	119%	124%	155%	210%	180%
XH	135%	136%	210%	288%	275%

**Table 4:** The values of $\eta_{2}$.

Concrete Group	$\mathrm{Strength}\;\mathrm{Index}\;\eta_{2}$				
	0 FTCs	10 FTCs	15 FTCs	20 FTCs	25 FTCs
XA	100% (30.7)	100% (24.17)	100% (22.13)	100% (17.17)	100% (16.2)
XB	6.24%	4.01%	2.08%	1.57%	1.48%
XE	5.26%	3.34%	2.98%	2.39%	1.31%
XF	6.45%	4.25%	3.03%	2.83%	2.06%
XG	7.44%	4.98%	3.22%	3.30%	2.66%
XH	8.41%	5.48%	4.37%	4.53%	4.06%

Note: The values in the bracelets are compressive strengths of cubic concrete, MPa.

**Table 5:** The ratio of increased shear strength by using C35 as the new concrete.

Concrete Group	New Concrete	The Shear Strengths of Interfaces/MPa				
		0 FTCs	10 FTCs	15 FTCs	20 FTCs	25 FTCs
XF	C30	1.981	1.027	0.6705	0.4867	0.3334
XI	C35	2.011	1.131	0.6915	0.5148	0.3901
The increased shear strength/MPa		0.03	0.104	0.021	0.0218	0.0567
The increasing ratio of shear strength		1.51%	10.13%	3.13%	5.77%	17.01%
Average of the ratio		7.51%				

**Table 6:** The physical properties of Portland cement.

Density (g/cm^3^)	Fineness (%)	Specific Surface Area (m^2^/g)	Stability	Setting Time (min)	
				Initial Setting	Final Setting
3.10	≤8.0	0.345	Qualified	226	279

**Table 7:** The chemical compositions of Portland cement (%).

CaO	SiO_2_	Al_2_O_3_	Fe_2_O_3_	MgO	SO_3_	Alkali	Ignition Loss
61.43	22.81	5.62	3.36	1.35	2.17	0.54	2.60

**Table 8:** Mix composition of concrete.

Strength Group	Water (kg/m^3^)	Cement (kg/m^3^)	Sand (kg/m^3^)	Aggregate (kg/m^3^)	W/C Ratio	Sand Ratio
C30	188	409	591	1200	0.46	33%
C35	188	470	539	1200	0.40	31%

**Table 9:** Outline of experimental cases.

Group Name	Old Concrete	New Concrete	Interface Type	Number of Specimens	Temperature Range
XB	C30	C30	No interface	3 × 5	−18~5 °C
XE	C30	C30	Interface without steel bar	3 × 5	−18~5 °C
XF	C30	C30	Reinforced by 8 mm steel bar	3 × 5	−18~5 °C
XG	C30	C30	Reinforced by 10 mm steel bar	3 × 5	−18~5 °C
XH	C30	C30	Reinforced by 12 mm steel bar	3 × 5	−18~5 °C
XI	C30	C35	Reinforced by 8 mm steel bar	3 × 5	−18~5 °C
XK	C30	C30	Reinforced by 8 mm steel bar	3 × 5	−12~5 °C
XM	C30	C30	Reinforced by 8 mm steel bar	3 × 5	−6~5 °C

**Table 10:** Roughness of the surface for old concrete (mm).

Group	XE	XF	XG	XH	XI	XK	XM
1-1	2.6	2.4	2.3	2.6	2.3	2.1	2.1
1-2	2.3	2.7	2.4	2.0	2.0	2.3	2.5
1-3	2.1	2.1	2.0	2.1	2.4	2.5	2.6
2-1	2.2	2.5	2.0	2.5	2.3	2.4	2.6
2-2	2.3	2.2	2.2	2.4	2.3	2.2	2.5
2-3	2.1	2.2	2.2	2.6	2.4	2.5	2.4
3-1	2.0	2.1	2.5	2.2	2.1	2.4	2.6
3-2	2.4	2.2	2.4	2.4	2.7	2.1	2.6
3-3	2.0	2.0	2.0	2.3	2.5	2.3	2.2
4-1	2.1	2.2	2.2	2.4	2.0	2.5	2.5
4-2	2.3	2.1	2.2	2.1	2.0	2.1	2.4
4-3	2.0	2.0	2.5	2.3	2.4	2.9	2.2
5-1	2.1	2.5	2.4	2.3	2.6	2.5	2.6
5-2	2.0	2.2	2.6	2.5	2.4	2.2	2.7
5-3	2.3	2.5	2.7	2.2	2.5	2.1	2.9
